# The prognostic role of the echocardiographic tricuspid annular plane systolic excursion/systolic pulmonary arterial pressure (TAPSE/sPAP) ratio and its relationship with NT-proANP plasma level in systemic sclerosis

**DOI:** 10.3389/fcvm.2022.1021048

**Published:** 2023-01-17

**Authors:** Maria Chiara Grimaldi, Edoardo Rosato, Adriano D’Angelo, Ernesto Cristiano, Simona Marchitti, Massimo Volpe, Speranza Rubattu, Antonella Romaniello

**Affiliations:** ^1^Department of Clinical and Molecular Medicine, Sapienza University of Rome, Rome, Italy; ^2^Department of Cardiovascular and Pneumological Sciences, Catholic University of Sacred Heart, Rome, Italy; ^3^Fondazione Policlinico Universitario Agostino Gemelli Istituto di Ricovero e Cura a Carattere Scientifico (IRCCS), Rome, Italy; ^4^Department of Translational and Precision Medicine, Sapienza University of Rome, Rome, Italy; ^5^Istituto di Ricovero e Cura a Carattere Scientifico (IRCCS) Neuromed, Pozzilli, Italy; ^6^San Raffaele Pisana Istituto di Ricovero e Cura a Carattere Scientifico (IRCCS), Rome, Italy; ^7^Division of Cardiology, Sant’Andrea Hospital, Rome, Italy

**Keywords:** right ventricular-arterial coupling, TAPSE/sPAP ratio, atrial natriuretic peptide (ANP), systemic sclerosis (scleroderma), pulmonary hypertension, pulmonary arterial hypertension (PAH), TAPSE/sPAP

## Abstract

**Background:**

The tricuspid annular plane systolic excursion/systolic pulmonary arterial pressure (TAPSE/sPAP) ratio is an echocardiographic estimation of the right ventricle to pulmonary artery (RV/PA) coupling, with a validated prognostic role in different clinical settings. Systemic sclerosis (SSc) patients without evident cardiovascular involvement frequently display subtle RV impairment. The amino-terminal atrial natriuretic peptide (NT-proANP) plasma level relates to SSc disease progression and mortality. We aimed to assess the prognostic value of the TAPSE/sPAP ratio and its relationship with NT-proANP plasma level in SSc patients without overt cardiovascular involvement.

**Methods:**

We retrospectively analysed 70 SSc consecutive patients, with no clinical evidence of cardiovascular involvement or pulmonary hypertension (PH), and 30 healthy controls (HC) in a retrospective, single-centre study. All SSc patients underwent recurrent clinical and echocardiographic assessments and NT-proANP plasma level was assessed at baseline. SSc-related cardiovascular events and deaths were extracted during a 6-year follow-up. The complete work-up for the diagnosis, treatment and management of PH performed along the 6 years of follow-up referred to the 2015 European Society of Cardiology guidelines.

**Results:**

Systemic sclerosis patients showed lower TAPSE/sPAP ratio at baseline compared to HC [SSc median value = 0.71 mm/mmHg, (IQR 0.62–0.88) vs. HC median value = 1.00 mm/mmHg, (IQR 0.96–1.05); *p* < 0.001]. Multivariable Cox analysis revealed TAPSE/sPAP ratio as an independent predictor for SSc-related cardiovascular events [HR = 3.436 (95% CI 1.577–7.448); *p* = 0.002] and mortality [HR = 3.653 (95% CI 1.712–8.892); *p* = 0.014]. The value of TAPSE/sPAP ratio < 0.7 mm/mmHg was identified as an optimal cut-off for predicting adverse outcomes (*p* < 0.001) by receiver operating characteristic (ROC) analyses. NT-proANP level significantly related to TAPSE/sPAP ratio (*r* = *0.52, p* < 0.001). TAPSE/sPAP ratio combined with NT-proANP showed an overall significant prognostic role in this SSc population, confirmed by Kaplan–Meier analysis (Log rank *p* < 0.001).

**Conclusion:**

The TAPSE/sPAP ratio, as an index of RV/PA coupling, is an affordable predictor of cardiovascular events and mortality in SSc and, combined with NT-proANP level, may improve the clinical phenotyping and prognostic stratification of SSc patients.

## 1. Introduction

Systemic sclerosis (SSc) is an autoimmune disease with a multisystem involvement characterised by widespread inflammation and microvascular alterations leading to ischemia and fibrosis (1, 2), particularly affecting the heart and lungs. Interstitial lung disease (ILD) and cardiovascular injuries are the most frequent complications, with a consequent severe impact on prognosis (3–5). Clinical evidence of cardiovascular disease may be found in 20–35% of patients with SSc, whereas the heart is affected in up to 80% of patients at post-mortem examination (6, 7).

Primary cardiac manifestations of SSc, due to myocardial fibrosis, impaired microcirculation and vascular wall remodelling, can range from myocardial disease, right and left heart failure (HF), conduction system abnormalities and arrhythmias, to coronary and pericardial disease and aggressive atherosclerosis. The involvement of the pulmonary circulation can lead to pulmonary arterial hypertension (PAH), with the consequent poorest prognosis (8). Despite a normal echocardiographic assessment, SSc patients often go through a right ventricle (RV) occult dysfunction and latent pulmonary vascular disease and hypertension, eventually revealed by stress echocardiography and haemodynamic evaluation (9, 10). Therefore, the active screening for an early diagnosis of sub-clinical cardiovascular and cardio-pulmonary impairment might be essential for timely treatment and adequate long-term management of SSc patients.

The mechanical and energetic efficiency of the cardiovascular system is determined by the dynamic interaction between the heart and vessels. The anatomical and functional link between the RV and pulmonary arterial (PA) vascular district underscores the importance to consider RV and PA as one combined unit. The ventricular-arterial coupling, defined by the end-systolic ventricular elastance (*Ees*) and arterial elastance (*Ea*) ratio is a reliable index of cardiovascular performance, derived from the invasive measurement of the pressure-volume relationship (11). In spite of the wide range of parameters assessing cardiac and pulmonary properties and functions, only the RV/PA coupling is a sensitive index of the cardio-pulmonary unit function (11, 12).

Among simpler surrogates that are therefore being validated, the tricuspid annular plane systolic excursion/systolic pulmonary arterial pressure (TAPSE/sPAP) ratio is an echocardiographic estimation of the RV/PA coupling, well approximating the invasive gold standard measurement (12, 13).

Previous studies demonstrated that the perturbation of the RV/PA coupling is a sensitive and prognostic parameter in different clinical settings. Likewise, TAPSE/sPAP ratio has been shown to have prognostic relevance in PAH (14, 15), HF with and without pulmonary hypertension (PH) (16, 17), pulmonary embolism (18) and PH in chronic lung disease (19). Based on its clinical relevance, the TAPSE/sPAP ratio was recently added to routine assessment for the diagnosis and management of PH according to the 2022 ESC/ERS guidelines (20). Instead, the role of the TAPSE/sPAP ratio in SSc patients without overt cardiovascular involvement has been so far poorly investigated.

Atrial natriuretic peptide (ANP), which participates in the cardiovascular system homeostasis, is synthesised in the heart as a pre-pro-hormone, subsequently cleaved into the biologically carboxy-terminal active peptide and the amino-terminal peptide (NT-proANP), which is a more stable circulating form (21). ANP is mainly released by atrial cardiomyocytes in response to hemodynamic stressors, such as pressure and volume overload. ANP release kinetic is highly triggered by the haemodynamic load applied to the right atrium and pulmonary circulation, mainly under exercise (22, 23). Simultaneously, the same hemodynamic triggers for ANP secretion contribute to impair the ventricular performance and arterial stiffness, promoting the RV/PA uncoupling.

Atrial natriuretic peptide is also secreted from the endothelium in response to vessel injuries and it exerts protective functions in an autocrine/paracrine manner (24, 25). NT-proANP plasma level predicted disease progression and mortality in SSc patients (26, 27). Since ANP arises in response to both the hemodynamic changes promoting RV/PA uncoupling and to the endothelial injury, a typical feature of SSc pathogenesis and progression, the increase of NT-proANP plasma level might be an early marker of the cardio-pulmonary unit involvement in asymptomatic SSc patients.

The present study aimed to investigate the prognostic value of the TAPSE/sPAP ratio as an index of RV/PA coupling and the relevance of TAPSE/sPAP ratio alone and combined with NT-proANP plasma level for the prediction of adverse outcomes in SSc patients.

## 2. Materials and methods

### 2.1. Study population

We analysed seventy consecutive patients (sixty-four women and six men) already diagnosed with SSc according to the criteria of the American College of Rheumatology/European League against Rheumatism (28, 29) in a single-centre, retrospective study, from 2014 to 2020, at Sant’Andrea Hospital, Rome, Italy. Exclusion criteria were cardiovascular, pulmonary and renal diseases, malignancies, coagulopathy, diabetes, dyslipidaemia, and smoking habit. Pregnant or breastfeeding women were excluded. Patients with severe SSc-related ILD were excluded (30, 31) (Supplementary Figure 1). Thirty healthy volunteers [healthy controls (HC)] were enrolled as the control group, with homologous characteristics compared to SSc patients.

All SSc patients underwent complete evaluation, including clinical examination, electrocardiogram, echocardiography, 6-min walking test, nailfold video capillaroscopy, high-resolution chest tomography (HRTC), pulmonary function test, diffusion lung carbon oxide (DLCO), both at baseline and yearly (or more when needed) for the 6-year follow-up (30, 31). DLCO and DLCO/VA assessment were performed using carbon monoxide and helium as tracer gas (26, 27).

According to the international recommendation (32–34) all SSc patients underwent comprehensive echocardiography yearly. All patients presented at least a mild tricuspid regurgitation, committed for sPAP estimation. Right heart catheterization was performed for the diagnosis of PH, in accordance with the 2015 ESC/ERS guidelines (35), when indicated.

Plasma NT-proANP level measurement was performed at baseline in all patients with SSc. The study protocol was approved by the locally appointed ethical committee and written informed consent was obtained from all participants, complied with the Declaration of Helsinki.

### 2.2. Outcomes

The primary composite outcome was the occurrence of all cardiovascular events referring to the cardiovascular manifestation of SSc, during the 6-year follow-up. The cardiovascular manifestations of SSc were defined as new diagnosis of PH (35), PAH (35), HF (36) requiring or not hospitalisation, arrhythmias and conduction disturbances, new onset of ischaemic heart disease and pericardial effusion. The secondary outcome was the SSc-related death occurring during the 6-year follow-up. Mortality for SSc was defined as death due to complications of ILD, PAH, PH, and HF.

### 2.3. Assessment of NT-proANP plasma level

Venous blood samples (3 ml) were collected from all SSc patients in EDTA tubes and centrifuged at 3000 rpm for 15 minutes. Measurements of the plasma level of NT-proANP were performed using an ELISA kit (Biomedica, Wien, Austria). The cut-off of normal value for NT-proANP was considered 2200 fmol/ml.

### 2.4. Echocardiography

Baseline echocardiographic data were acquired using commercially available equipment and standard views, in accordance with the international guidelines (35–38). For each patient, simple parameters and derived measures were obtained and recorded at baseline and during the 6-year follow-up. All echocardiographic data were performed by a board-certified cardiologist echocardiographer and independently reviewed by a board-certified echocardiographer blinded to the outcomes. Right ventricular outflow tract (RVOT) diameter was obtained in 2D long-axis parasternal view as a linear dimension measured from the anterior RV wall to the interventricular septal-aortic junction. RVOT Doppler acceleration time was measured from the beginning of the flow to the peak flow velocity. Proximal diameter of the main pulmonary artery was measured in 2D short axis view to evaluate dilatation. TAPSE was measured on M-mode images as the difference in RV basal motion from peak systole to end-diastole. RV and left ventricle (LV) diameters were measured at end-diastole in the four-chamber view to calculate RV/LV ratio. The tricuspid regurgitation (TR) severity was classified as mild, moderate, or severe. The maximal tricuspid regurgitation velocity (TRV) by continuous-wave Doppler was used to derive the RV–right atrial (RA) pressure gradient by the simplified Bernoulli equation. RA pressure (RAP) was estimated based on the inferior vena cava (IVC) end-expiratory diameter and collapsibility at the end-inspiratory phase, as recommended (38). The peak sPAP was assessed by adding the estimated RAP to the systolic *trans*-tricuspid pressure gradient. The TAPSE/sPAP ratio was then calculated for all patients and healthy subjects.

### 2.5. Statistical analysis

Continuous distributed variables were represented as median and interquartile range (IQR) and categorical data were expressed as frequencies. Data were analysed for normality by the coefficient of kurtosis and by the Shapiro–Wilk test. Appropriate statistical parametric and non-parametric (Student’s *t*, Mann–Whitney *U*, *X*^2^) tests were used in the different analyses. Receiver operating characteristic (ROC) analyses and the Youden Index were performed to test the TAPSE/sPAP ratio in the prediction of outcomes and to determine the optimal cut-off value. Univariable and multivariable Cox regression models were performed to assess the prognostic relevance of different variables to outcomes.

To avoid collinearity and overfitting, the multivariable Cox model included the following continuous variables: age, DLCO, TAPSE/sPAP ratio, and NT-proANP for both primary and secondary outcomes.

A reduced model by backward stepwise multivariable regression analysis was used to detect independent predictors. Linear correlation was assessed by Spearman’s coefficient.

Kaplan–Meier analysis was used to evaluate overall survival and events recurrence, with the log rank test for comparison. The subjects were censored after any of the composite outcomes were reached.

To evaluate the incremental value of TAPSE/sPAP and NT-proANP plasma level, two models were compared: model 1 as TAPSE/sPAP alone and model 2 as TAPSE/sPAP ratio combined with NT-proANP. Global *X*^2^ and incremental significance of these models were calculated. The strength of independent predictors was compared using the concordance index (C-index). Akaike information criterion (AIC) method was added for the comparison of model fit (Supplementary Table 1).

A two-sided *p*-value < 0.05 was considered statistically significant in all analyses. Coefficients obtained from regression models were expressed in terms of hazard ratio (HR) with 95% confidence intervals (CI). All statistical analyses were performed using SPSS software (version 27.0, IBM) and Prism GraphPad (version 8.1).

## 3. Results

### 3.1. Baseline characteristics, outcomes, and TAPSE/sPAP ratio distribution

Seventy SSc patients (sixty-four women and six men) without overt cardiovascular involvement were enrolled in this study. The majority of the study population (67%) presented with the limited cutaneous subtype of SSc while the remaining 33% manifested the diffuse cutaneous systemic scleroderma subtype. Demographic, clinical, and echocardiographic characteristics of the SSc population and controls are reported in Table 1.

**TABLE 1:** Baseline demographic, clinical, and echocardiographic characteristics of the SSc population and healthy individuals (HC).

	SSc (n = 70)	HC (n = 30)	*p*
Female, *n* (%)	64 (90)	27 (90)	0.132*
Age, years	59 (46–69)	55 (45–67)	0.105**
BMI, Kg/m^2^	23 (21–26)	23 (21–26)	0.113**
**SSc disease characteristics**			
Disease duration, years	8 (5–15)	n/a	–
dcSSc *n* (%)	23 (33)	n/a	–
lcSSc *n* (%)	47 (67)	n/a	–
Digital ulcers history, *n* (%)	24 (34)	n/a	–
ILD, *n* (%)	14 (20)	n/a	–
SSc-specific autoantibodies, *n* (%)			–
Anti-topoisomerase I	21 (30)	n/a	
Anti-centromere	42 (60)	n/a	
None	7 (10)	n/a	
Capillaroscopic pattern, *n* (%)			–
Early	22 (31.4)	n/a	
Active	22 (31.4)	n/a	
Late	26 (37.2)	n/a	
Pulmonary function test			–
FVC, % of predicted value	94.5 (77.5–110)	n/a	
DLCO, % of predicted value	71 (48.9–82.4)	n/a	
NT-proANP (fmol/ml)	1799 (1384–2820)	n/a	–
**Echocardiographic variables**			
RVOT diameter (mm)	26 (24–35)	25 (24–28)	**0**.049**
RV/LV ratio, *n* (%)			
<1	49 (70)	25 (83)	**<0**.001***
1	20 (28)	5 (17)	**<0**.001***
>1	1 (2)	0	**<0**.001***
LV eccentricity index (end-diastole)	1.0 (0.9–1.1)	1.0 (0.9–1.0)	ns
TR, *n* (%)			
Mild	46 (66)	25 (83)	**<0**.001***
Moderate	22 (31)	5 (17)	**<0**.001***
Severe	2(3)	0	**<0**.001***
TRV (cm/s)	2.7 (2.3–3.1)	2.1 (1.9–2.2)	**<0**.001**
RV-RA pressure gradient (mmHg)	21 (22–30)	18 (16–22)	**<0**.001**
sPAP (mmHg)	31 (27–37)	25 (22–27)	**<0**.001**
TAPSE (mm)	23 (21–26)	25 (21–28)	**0**.041**
RV S’ (cm/s)	12.5 (11–15)	14 (12–17)	**0**.043**
TAPSE/sPAP (mm/mmHg)	0.71 (0.62–0.88)	1.00 (0.96–1.05)	**<0**.001**
IVC end-expiratory diameter (mm)	17 (14–21)	16 (14–20)	0.129**
RA area (cm^2^)	13 (11–20)	12 (10–17)	0.051**
RVOT Doppler acceleration time (msec)	126 (106–140)	136 (110–155)	**0**.039**
Pulmonary artery diameter (mm)	24 (22–26)	24 (21–25)	ns
LV ejection fraction, %	60 (58–70)	60 (59–70)	0.875***
LV diastolic function, *n* (%)			
Normal	56 (80)	27 (90)	**<0**.001***
Diastolic dysfunction	14 (20)	3 (10)	**<0**.001***

Data are presented as median and inter-quartile range or percentage, as appropriate. Statistical tests used to assess differences between subgroups: *Student’s *t*-test, **Mann–Whitney *U*-test; ***X^2^-test. The *p*-values < 0.05 are highlighted in bold.

BMI, body mass index; dcSSc, diffuse cutaneous systemic sclerosis; DLCO, diffusing capacity of the lung for carbon monoxide; FVC, forced vital capacity; ILD, interstitial lung disease; IVC, inferior vena cava; lcSSc, limited cutaneous systemic sclerosis; LV, left ventricle; NT-proANP, amino-terminal atrial natriuretic peptide; sPAP, systolic pulmonary arterial pressure; RA, right atrium; RV, right ventricle; RV S’, systolic annular tissue velocity of the lateral tricuspid annulus; RVOT, right ventricular outflow tract; SSc, systemic sclerosis; TAPSE, tricuspid annular plane systolic excursion; TR, tricuspid regurgitation; TRV, tricuspid regurgitant velocity.

During the 6 years of follow-up, thirty-six patients (51% of the study population) experienced an SSc-related cardiovascular event. We reported six new PAH diagnoses (17%) and seven new diagnoses of right HF (19%), four of them requiring hospitalisations. Ten patients (28%) experienced arrhythmias (seven cases of documented supraventricular tachycardia, two cases of documented ventricular tachycardia, and one case of atrioventricular block requiring a pacemaker implantation). Seven patients (19%) had angina without evidence of coronary artery disease and six patients (17%) experienced more than mild pericardial effusion, one of them requiring pericardiocentesis (Supplementary Figure 2A).

The diagnosis of PH and PAH was performed according to the current 2015 ESC/ERS guidelines (35), during the 6-year follow-up. However, by applying the last 2022 ESC/ERS guidelines and the new cut-off for the diagnosis of PH (20), our patients fulfil the inclusion criteria and there would be no difference on the primary outcome.

Out of the seventy SSc patients included in our study, we reported sixteen SSc-related deaths (23%): nine (56%) were attributed to ILD complications, four (25%) to PAH progression and three (19%) to HF-related fatalities (Supplementary Figure 2B). These outcomes are concordant with those reported in the international SSc registries (3–5).

Systemic sclerosis patients showed lower values of the TAPSE/sPAP ratio compared to controls at baseline [SSc median value = 0.71 mm/mmHg, (IQR 0.62–0.88) vs. HC median value = 1.00 mm/mmHg, (IQR 0.96–1.05); *p* < 0.001] (Table 1).

**FIGURE 1:**
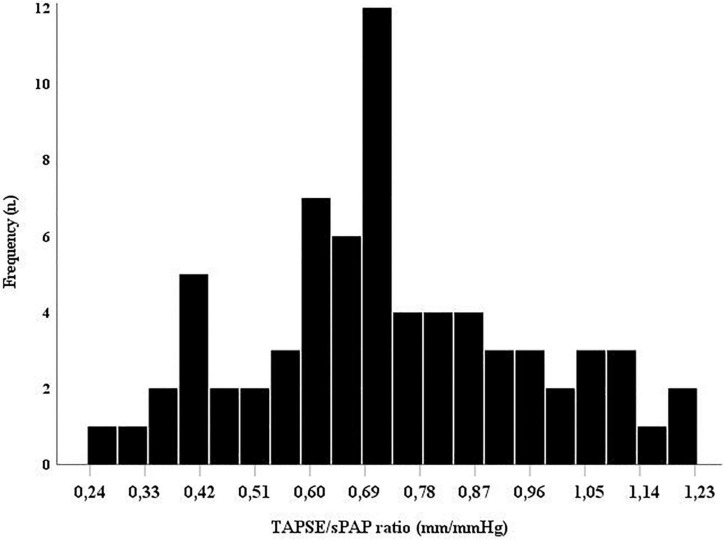
The TAPSE/sPAP ratio distribution in this population of systemic sclerosis (SSc) patients. Histogram showing the overall TAPSE/sPAP ratio distribution in the SSc population at baseline. sPAP, systolic pulmonary arterial pressure; TAPSE, tricuspid annular plane systolic excursion.

The distribution of the TAPSE/sPAP ratio in the SSc population at baseline is reported in Figure 1.

The median value of the TAPSE/sPAP ratio at baseline was significantly lower in SSc patients facing primary outcome vs. SSc patients without cardiovascular events [0.62 (0.47–0.74) vs. 0.77 (0.71–0.95) mm/mmHg; *p* < 0.001] on 6-year follow-up.

According to the secondary outcome, the baseline TAPSE/sPAP ratio was significantly lower in patients who died for SSc-related causes in comparison with survived patients [0.51 (0.41–0.62) vs. 0.76 (0.68–0.95) mm/mmHg; *p* < 0.001].

### 3.2. Receiver operating characteristic (ROC) analysis for the TAPSE/sPAP ratio and outcomes

**FIGURE 2:**
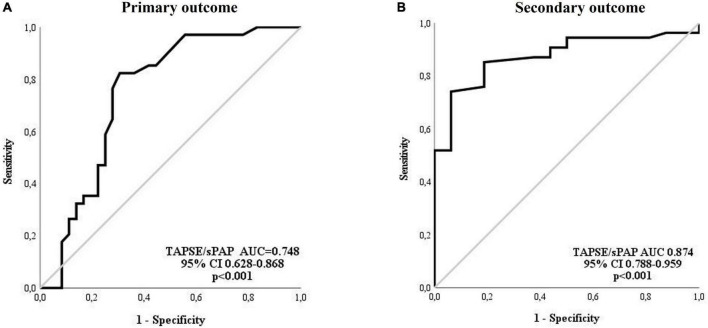
Receiver operating characteristic (ROC) analysis for the TAPSE/sPAP ratio and outcomes. **(A)** Primary outcome: The TAPSE/sPAP ratio had an AUC of 0.748 (95% CI 0.628–0.868; *p* < 0.001) for the prediction of cardiovascular events (sensitivity 82% and specificity 70%). **(B)** Secondary outcome: The TAPSE/sPAP ratio had an AUC of 0.874 (95% CI 0.788–0.959; *p* < 0.001) for the prediction of death (sensitivity 70% and specificity 94%). AUC, area under the curve; CI, confidence interval; sPAP, systolic pulmonary arterial pressure; TAPSE, tricuspid annular plane systolic excursion.

In accordance with the ROC analysis, the value of TAPSE/sPAP ratio < 0.7 mm/mmHg resulted as a significant predictor of both primary [sensitivity = 82%, specificity = 70%, AUC 0.748 (95% CI 0.628–0.868); *p* < 0.001] and secondary [sensitivity = 70%, specificity = 94%, AUC 0.874 (95% CI 0.788–0.959); *p* < 0.001] outcomes (Figures 2A, B, respectively).

Patients were dichotomised into high or low TAPSE/sPAP ratio assuming the value of 0.7 mm/mmHg as the threshold. A significantly higher proportion of all reported cardiovascular events (75%) and deaths (100%) occurred in patients with a low TAPSE/sPAP ratio (<0.7 mm/mmHg) compared to patients with a high TASPE/sPAP ratio (≥0.7 mm/mmHg) during the 6 years of follow-up (Supplementary Figures 3A, B).

### 3.3. Univariable and multivariable Cox analyses and outcomes

In the univariable Cox regression analysis, the TAPSE/sPAP ratio, as a continuous variable, was significantly associated with both primary [HR 4.616 (95% CI 2.240–9.514); *p* < 0.001] and secondary outcomes [HR 4.048 (95% CI 3.177–8.262); *p* = 0.002] (Table 2).

**TABLE 2:** Univariable and multivariable Cox regression analyses for independent predictors of outcomes.

Primary outcome						
	Univariable analysis			Multivariable analysis*		
Variables	HR	95% CI	*p*	HR	95% CI	*p*
Age (years)	1.032	1.007–1.058	**0**.**013**	0.99	0.953–1.028	0.604
Disease duration (years)	1.005	0.965–1.047	0.799			
Anti-topoisomerase I	0.772	0.378–1.576	0.477			
DLCO (% of predicted value)	0.981	0.966–0.996	**0**.**012**	0.984	0.967–1.000	0.056
TAPSE (mm)	0.936	0.846–1.036	0.201			
sPAP (mmHg)	1.061	1.033–1.089	**<0**.**001**			
NT-proANP (fmol/ml)	3.455	1.738–6.866	**<0**.**001**	2.461	1.029–5.887	**0.043**
TAPSE/sPAP ratio (mm/mmHg)	4.616	2.240–9.514	**<0**.**001**	3.709	1.602–8.588	**0.002**
**Reduced model** **						
TAPSE/sPAP ratio (mm/mmHg)	–	–	–	3.436	1.577–7.448	**0.002**
NT-proANP (fmol/ml)	–	–	–	2.196	1.046–4.608	**0.038**
**Secondary outcome**						
Age (years)	0.072	1.022–1.119	**0**.**005**	1.016	0.941–1.097	0.687
Disease duration (years)	1.013	0.958–1.071	0.647			
Anti-topoisomerase I	0.536	0.173–1.661	0.28			
DLCO (% of predicted value)	0.962	0.941–0.984	**<0**.**001**	0.976	0.951–1.002	0.069
TAPSE (mm)	0.858	0.738–0.998	**0**.**048**			
sPAP (mmHg)	1.076	1.042–1.112	**<0**.**001**			
NT-proANP (fmol/ml)	9.548	2.714–3.586	**<0**.**001**	4.188	1.028–7.063	**0.046**
TAPSE/sPAP ratio (mm/Hg)	4.048	3.177–8.262	**0**.**002**	3.927	1.498–9.549	**0.02**
**Reduced model** **						
TAPSE/sPAP ratio (mm/mmHg)	–	–	–	3.653	1.712–8.892	**0.014**
NT-proANP (fmol/ml)	–	–	–	4.418	1.217–6.032	**0.024**

*To avoid collinearity and overfitting, the multivariable analysis included the following continuous variables: Age, DLCO, TAPSE/sPAP, and NT-proANP.

**Reduced model by backward stepwise multivariable Cox regression analysis. Multivariable model is adjusted for clinical covariates. The *p*-values < 0.05 are highlighted in bold. CI, confidence interval; DLCO, diffusing capacity of the lung for carbon monoxide; HR, hazard ratio; NT-proANP, amino-terminal atrial natriuretic peptide; sPAP, systolic pulmonary arterial pressure; TAPSE, tricuspid annular plane systolic excursion.

In the multivariable analysis, the TAPSE/sPAP ratio showed a significant independent association with both primary [HR 3.709 (95% CI 1.602–8.588); *p* = 0.002] and secondary [HR 3.927 (95% CI 1.498–9.549); *p* = 0.020] outcomes (Table 2).

Also, DLCO showed a significant correlation with mortality in the univariable analysis [HR 0.962 (95% CI 0.941–0.984); *p* < 0.001], but not in the multivariable (Table 2). Of note, the presence of anti-topoisomerase I antibodies, linked to a poor prognosis and SSc-related mortality, was not significant in our cohort of patients (*p* = 0.280) (Table 2).

The duration of the disease was not significant according to cardiovascular events and mortality in our population.

Additional stepwise multivariable regression analysis confirmed both the TAPSE/sPAP ratio and NT-proANP plasma level as independent prognostic factors for both primary [HR 3.436 (95% CI 1.577–7.448); *p* = 0.002 and HR 2.196 (95% CI 1.046–4.608); *p* = 0.038, respectively] and secondary outcomes [HR 3.653 (95% CI 1.712–8.892); *p* = 0.014 and HR 4.418 (95% CI 1.217–6.032); *p* = 0.024, respectively] (Table 2).

### 3.4. NT-proANP plasma level distribution and its relationship with the TAPSE/sPAP ratio

The overall median value of NT-proANP plasma level in this cohort was 1799 fmol/ml (1384–2820 fmol/ml). NT-proANP level was significantly higher in SSc patients experiencing cardiovascular complications vs. SSc patients free from events, and in patients who died for SSc-related causes vs. survived population, consistently with previous evidence (26, 27).

**FIGURE 3:**
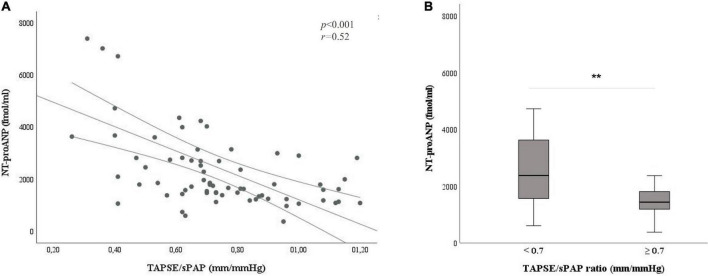
Relation between the TAPSE/sPAP ratio and NT-proANP plasma level. **(A)** Correlation between the TAPSE/sPAP ratio and NT-proANP plasma level (*r* = 0.52, *p < 0.001). **(B)** NT-proANP plasma level median value in patients with TAPSE/sPAP ratio < 0.7 vs. ≥ 0.7 mm/mmHg. [2735 (1815–4023) vs. 1511 (1221–1821) fmol/ml; ^**^p < 0.001]. The cut-off value of TAPSE/sPAP ratio 0.7 mm/mmHg was derived from the ROC curves and the Youden Index. NT-proANP, amino-terminal atrial natriuretic peptide; sPAP, systolic pulmonary arterial pressure; ROC, receiver operating characteristic; TAPSE, tricuspid annular plane systolic excursion; *Spearman’s correlation coefficient; ^**^Student’s *t*-test.

In the present work, we aimed at exploring the interaction between TAPSE/sPAP ratio and NT-proANP plasma level in SSc patients. A moderate but significant correlation was observed between the two parameters [*r* = 0.52, *p* < 0.001] (Figure 3A).

NT-proANP plasma level was significantly increased in SSc patients with the TAPSE/sPAP ratio < 0.7 mm/mmHg compared with patients with the TAPSE/sPAP ratio ≥ 0.7 mm/mmHg [2735 (1815–4023) vs. 1511 (1221–1821) fmol/ml; *p* < 0.001] (Figure 3B).

**FIGURE 4:**
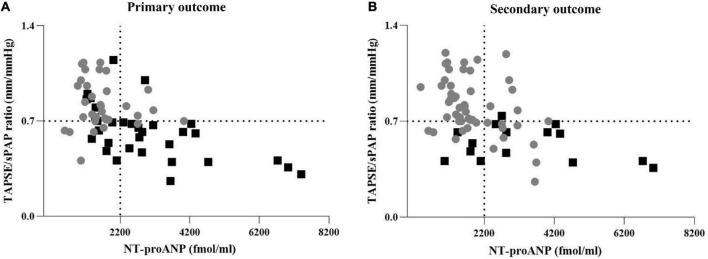
Distribution of the TAPSE/sPAP ratio and NT-proANP plasma level according to outcomes. **(A)** Distribution of TAPSE/sPAP ratio and NT-proANP plasma levels according to the primary outcome. Grey points: SSc patients free from cardiovascular events. Black squares: Patients with SSc-related cardiovascular events. **(B)** Distribution of TAPSE/sPAP ratio and NT-proANP plasma levels according to the secondary outcome. Grey points: Survived SSc patients. Black squares: SSc-related deaths. The TAPSE/sPAP ratio value of 0.7 mm/mmHg and NT-proANP value of 2,200 fmol/ml were set as thresholds. The cut-off value of TAPSE/sPAP ratio < 0.7 mm/mmHg was derived from ROC curves and the Youden Index. NT-proANP, amino-terminal atrial natriuretic peptide; sPAP, systolic pulmonary arterial pressure; ROC, receiver operating characteristic; TAPSE, tricuspid annular plane systolic excursion.

The distribution of the TAPSE/sPAP ratio and NT-proANP plasma levels, according to both primary and secondary outcomes, is shown in Figures 4A, B, respectively.

**FIGURE 5:**
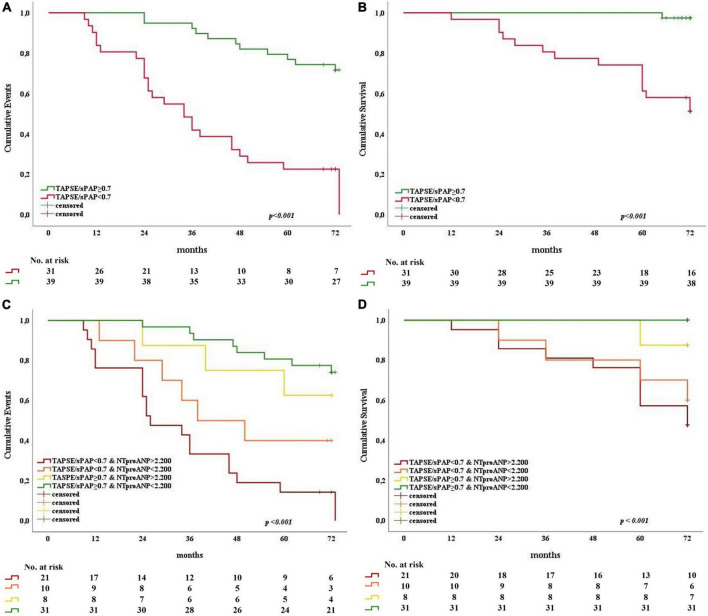
Kaplan–Meier analysis for primary and secondary outcomes as a function of the TAPSE/sPAP ratio alone and combined with NT-proANP plasma level in SSc patients. Patients were dichotomised at the TAPSE/sPAP ratio value of 0.7 mm/mmHg and NT-proANP value of 2200 fmol/ml. Stratifications by TAPSE/sPAP ratio alone **(A,B)** and combined with high and low NT-proANP plasma levels **(C,D)** showed significantly increased adverse outcomes (*n* = 70; Log-rank *p* < 0.001). **(A)** TAPSE/sPAP ratio and primary outcome (*X*^2^ 25.182; Log rank *p* < 0.001). **(B)** TAPSE/sPAP ratio and secondary outcome (*X*^2^ 20.783; Log rank *p* < 0.001). **(C)** TAPSE/sPAP ratio combined with NT-proANP and primary outcome (*X*^2^ 30.933; Log rank *p* < 0.001). **(D)** TAPSE/sPAP ratio combined with NT-proANP and secondary outcome (*X*^2^ 22.473; Log rank *p* < 0.001). NT-proANP, amino-terminal atrial natriuretic peptide; sPAP, systolic pulmonary arterial pressure; TAPSE, tricuspid annular plane systolic excursion.

The prognostic significance of the TAPSE/sPAP ratio was confirmed by Kaplan–Meier analysis for both primary and secondary outcomes (Log rank *p* < 0.001). SSc patients with TAPSE/sPAP ratio ≥ 0.7 mm/mmHg showed a significantly lower rate of cardiovascular events recurrence (Figure 5A) and better survival (Figure 5B) compared with SSc patients with TAPSE/sPAP ratio < 0.7 mm/mmHg. Also, our data showed a significant prognostic value of the TAPSE/sPAP ratio combined with NT-proANP plasma level in this SSc population (Figures 5C, D).

## 4. Discussion

In the present study, we provide evidence that SSc patients without evident cardiovascular involvement show a lower median value of the TAPSE/sPAP ratio, as an index of RV/PA uncoupling, compared to healthy subjects. The NT-proANP plasma level showed a correlation with the TAPSE/sPAP ratio. The TAPSE/sPAP ratio alone and in combination with NT-proANP plasma levels correlate with worse outcomes in our SSc population.

Cardiac involvement is common in SSc, even in the absence of clinical manifestations. Endomyocardial biopsies from SSc patients free from any cardiovascular complications (HF, PH and PAH, LV hypertrophy, arrhythmias, pericardial, and myocardial diseases) demonstrated an abnormal collagen deposition which is the distinguishing histological feature of SSc (39). Previous studies showed that patchy myocardial fibrosis and coronary microvascular dysfunction affect both RV and LV performance, impairing systolic (contractile) and diastolic (filling) functions (40, 41) in SSc. Echocardiographic studies showed that RV diastolic dysfunction can be detected even in the presence of normal sPAP, due to SSc *per se* and to the associated pulmonary vasculopathy (9). The immune abnormalities, the persistent interstitial inflammation, the increased extracellular matrix deposition together with the abnormal collagen cross-linking, the severe microangiopathy, characterised by arteriolar hyperplasia of the heart and lungs might explain, at least in part, the intrinsic myocardial impairment and the pulmonary vasculopathy contributing to RV/PA uncoupling in SSc patients.

The RV/PA uncoupling can be considered the driving cause of RV maladaptation and eventually RV failure. Previous echocardiographic and haemodynamic studies suggest that RV dysfunction is more severe in SSc patients (regardless of the presence of PH and ILD) compared to idiopathic PAH and it is also due to a greater afterload mismatch. This condition is related to the intrinsic myocardial dysfunction, decreased pulmonary arterial compliance, latent PH, leading to RV/PA uncoupling, RV negative remodelling and failure. This evidence underlines the poor survival of SSc patients with PAH (9).

According to the pathophysiology of SSc, characterised by endothelial injury, fibrosis and vascular remodelling of the heart and lungs, it appears mandatory to investigate the RV and the pulmonary vascular circuit as one combined physical unit. To this aim, the RV/PA coupling assessment better describes the functional state and efficiency of the cardio-pulmonary unit and can detect an impairment even when other indices are still preserved. The gold standard method for the quantification of RV/PA coupling relies on the invasive measurement of the *Ees/Ea* ratio calculated from a pressure-volume relationship, based on single-beat or multi-beat methods (11, 12, 42, 43). However, these measurements are invasive, technically, and costly demanding. A non-invasive gold standard approach for the evaluation of coupling has been developed based on RV volumes assessment by cardiac magnetic resonance (CMR) imaging (44–46).

The traditional echocardiographic evaluation of RV function and afterload may not fully detect the critical aspects of RV and PA interaction and dysfunction, especially in SSc population. Indeed, the echocardiographic TAPSE/sPAP ratio is a simpler, non-invasive, surrogate index of the RV/PA coupling and is more feasible in routine clinical practice (11).

The normal values of the *Ees/Ea* ratio range between 1.5 and 2.0 (11) whereas a normal TAPSE/sPAP ratio is about 1.0 in healthy subjects, ranging from 0.8 and 1.3 in healthy populations described in different studies (47–49).

The RV/PA coupling expresses a considerable reserve, since the *Ees/Ea* ratio must decrease from normal values to less than 0.8 before substantial RV maladaptation and clinically evident symptoms occur. Previous studies about the invasive measurements of RV/PA coupling demonstrated that a value of the *Ees/Ea* ratio < 0.7 in patients with PH was associated with clinical worsening (12). It is well known that patients with PAH receiving targeted therapies may remain stable for several years unless a severe uncoupling precipitates hemodynamic and clinical conditions. In this setting, RV/PA uncoupling is a sensitive marker of disease progression (46).

In this study, SSc patients showed a lower median value of TAPSE/sPAP ratio in comparison to controls (0.71 vs. 1.00 mm/mmHg) at baseline. A TAPSE/sPAP ratio < 0.7 mm/mmHg might reflect the intrinsic myocardial dysfunction, decreased pulmonary arterial compliance and increased vulnerability to ischemia, all typical features of the SSc disease.

Our original findings introduce the TAPSE/sPAP ratio as an independent prognostic echocardiographic parameter in patients with SSc. Although several indicators of RV function have been related to adverse events and survival (50), our data demonstrated that the TAPSE/sPAP ratio might be more specific and accurate, as it better reflects the RV performance and the load-dependent status of the pulmonary circulation.

Our previous studies demonstrated that NT-proANP plasma level correlated with vascular complications such as digital ulcers, worsening of PH and new onset of PAH in SSc patients (26) and it significantly predicted death due to the disease (27). In the present study, the NT-proANP moderately correlated with the TAPSE/sPAP ratio. Despite the lack of a strong statistical correlation coefficient (*r* = 0.52; *p* < 0.001), this evidence still carries a clinical and pathophysiological significance, linking plasma NT-proANP elevation to RV/PA uncoupling in SSc population.

The ANP release pattern is highly dependent on the hemodynamic stress triggers applied to the right atrium and pulmonary circulation, mainly under exertion (22, 23). For these reasons, the NT-proANP might represent a potential accurate biomarker related to the right atrial stress and impaired pulmonary hemodynamic.

Notably, the analysis of NT-proBNP level in this cohort did not reveal a statistically significant association with the TAPSE/sPAP ratio and outcomes.

Pulmonary involvement in SSc consists more often of interstitial fibrosis and pulmonary vascular disease, potentially leading to PH and PAH. Overall, chronic inflammation and consequent endothelial dysfunction are the main causes of SSc-related pulmonary injury, also contributing to ANP release (51). According to the pathophysiological link of this cardiac hormone with the endothelial homeostasis, pulmonary injury and hemodynamic stress contributing to ventricular-arterial uncoupling, NT-proANP is expected to behave as an appropriate biomarker for SSc-related cardiac, vascular, and pulmonary complications.

As the main original observation of our study, we report the first evidence that the TAPSE/sPAP ratio alone and in combination with NT-proANP plasma level are significant predictors of adverse outcomes due to SSc.

## 5. Conclusion

As far as our knowledge goes, the present study is the first one investigating the prognostic role of TAPSE/sPAP ratio, alone and in combination with NT-proANP plasma level, in an SSc population free from clinically evident cardiovascular manifestations. We provide evidence that the combined echocardiographic ratio of RV function (TAPSE) to afterload (sPAP) is an affordable predictor of adverse outcomes in SSc. This parameter can improve the clinical phenotyping and risk assessment of patients that may suffer from clinical deterioration and consequent worse prognosis on long-term follow-up. Importantly, the combination of TAPSE/sPAP ratio with NT-proANP plasma level, a known predictive biomarker in SSc, may open new perspectives in the clinical assessment and prognostic stratification of SSc patients.

### 5.1. Clinical implementations

The expression “the sooner, the better” is a paradigm in medicine, which suggests that an early diagnosis allows for a better outcome. SSc provides a perfect example of the need for useful tools to identify early subclinical cardiac impairment and improve survival through timely intervention.

Among the connective tissue diseases, SSc demonstrates a unique physiopathology and clinical phenotype with the poorest survival. Although screening and therapeutic strategies for SSc patients significantly evolved, important clinical needs remain unmet, particularly the availability of more appropriate tests and biomarkers for the screening and monitoring of early cardiovascular involvement. Echocardiographic measurements and biomarkers are already included in diagnostic algorithms and prognostic risk scores, but none of them strongly refers to the right ventricular function and the RV-PA unit. Also, a hormonal indicator of both pulmonary vascular and cardiac remodelling and impaired hemodynamic, like ANP, could better fit the need for an early diagnosis. In this scenario, the TAPSE/sPAP ratio and plasma NT-proANP level seem to be appropriate parameters to be used in the early clinical assessment of SSc patients, due to their greater sensitivity, specificity, and prognostic relevance. We provide the first evidence that the TAPSE/sPAP ratio, both when used alone and in combination with NT-proANP plasma level, appears the most suitable parameter for the clinical and prognostic assessment of SSc patients.

### 5.2. Limitations

First, this was a retrospective and single-centre study including a small moderately heterogeneous cohort of SSc patients. However, we believe it could represent a useful pilot study to validate the TAPSE/sPAP ratio and NT-proANP as early markers of cardiovascular involvement in a wider population of SSc patients. Further investigation is needed to strengthen and better explain the pathophysiological relationship between the TAPSE/sPAP ratio and NT-proANP plasma level and its clinical impact.

Moreover, our SSc population included a small number of male patients, in accordance with the typical epidemiological features of the disease. Patients involved in our study presented different clinical SSc subtypes and disease duration at the time of enrolment.

The echocardiographic evaluation of RV function has some limitations. Although TAPSE is a common estimate of RV function, it does not reflect the whole RV performance since longitudinal RV function may differ from radial or apical RV contractility. To this aim, the speckle tracking-derived measurement of myocardial deformation could be an accurate tool for the early detection of subclinical myocardial dysfunction, with the potential to provide additional insights into the course of SSc cardiovascular disease (52, 53). On the other hand, TAPSE/sPAP ratio is a simple, well-validated quantitative parameter easy to apply in clinical practice. Furthermore, a large validation of the TAPSE/sPAP ratio with an invasive assessment of the *Ees/Ea* ratio is lacking in this SSc population.

## Data availability statement

The raw data supporting the conclusions of this article will be made available by the authors, without undue reservation.

## Ethics statement

The studies involving human participants were reviewed and approved by the Ethical Committee of the Sant’Andrea Hospital. The patients/participants provided their written informed consent to participate in this study.

## Author contributions

MCG performed the study conception, data management and interpretation, statistical analyses, first draft, revisions, and proofread of the manuscript. MCG, AR, and SR designed the research, contributed to suggestions and critical reading of the manuscript, and edited the final version. SR provided funding support. SR and AR collected biological materials. SM and SR processed biological materials. AR, MCG, ER, AD’A, and EC performed the selection and screening of the patients. All authors read and approved the submitted version.
