# Supplementary material for: The prognostic role of the echocardiographic tricuspid annular plane systolic excursion/systolic pulmonary arterial pressure (TAPSE/sPAP) ratio and its relationship with NT-proANP plasma level in systemic sclerosis
Source: Front Cardiovasc Med. 2023 Jan 17;9:1021048. doi: 10.3389/fcvm.2022.1021048 (PMC9887033; doi:10.3389/fcvm.2022.1021048)
Supplement: Supplementary file 1 [file Data_Sheet_1.pdf]

Supplementary Material

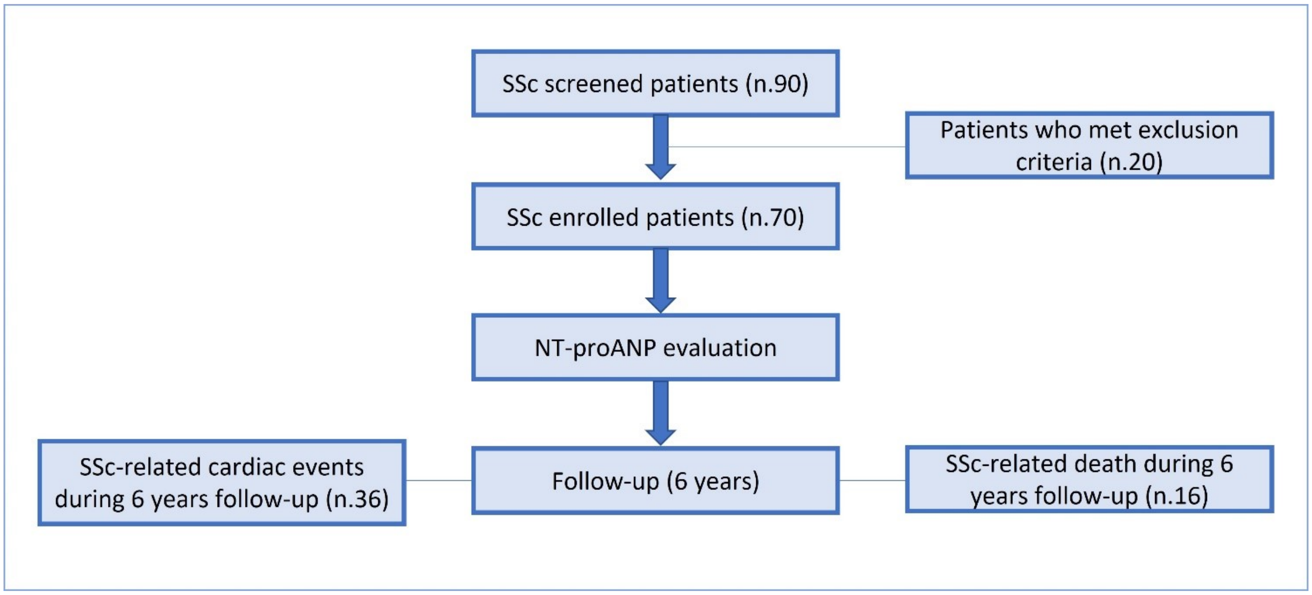

Supplementary Figure 1. Study Flow Chart.
NT-proANP: amino-terminal atrial natriuretic peptide; SSc, systemic sclerosis.

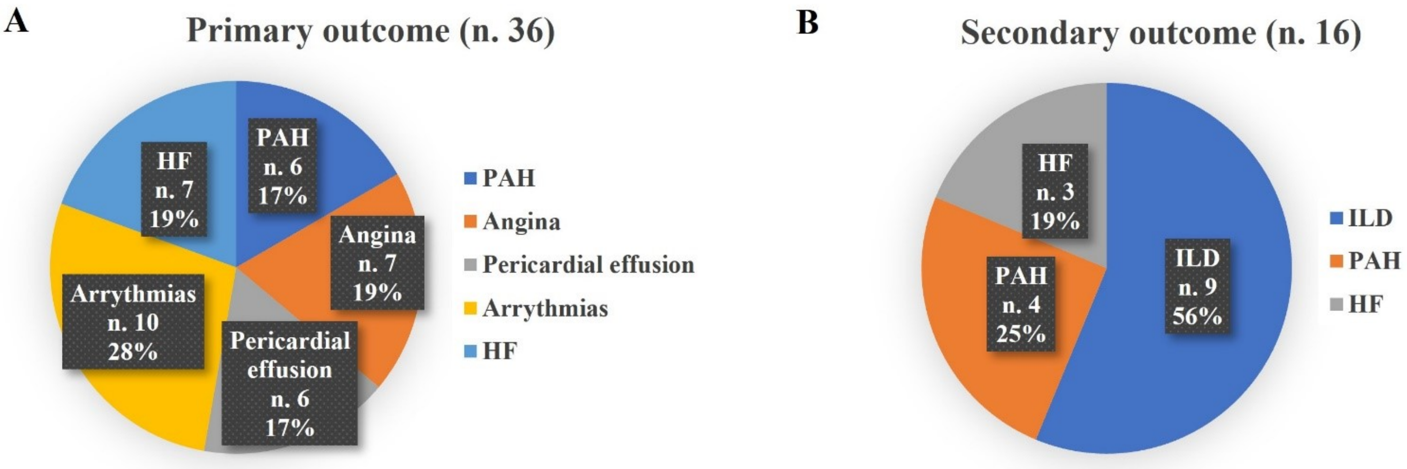

Supplementary Figure 2. Percentage of cumulative events for primary (A) and secondary (B) outcomes.
(A) During the 6 years of follow-up, 36 of 70 patients in the study population experienced an SSc-related cardiac event: 6 new PAH diagnoses (17%); 7 new diagnoses of right HF (19%); 10 cases of arrhythmias (28%); 7 diagnoses of angina without evidence of coronary artery disease (19%) and 6 diagnoses of more than mild pericardial effusion (17%). **(B)** Of the 70 SSc patients included in our study, 16 SSc-related deaths were reported: 9 were attributed to ILD complications (56%); 4 to PAH progression (25%) and 3 to HF-related fatalities (19%).
HF, heart failure; ILD interstitial lung disease; PAH, pulmonary arterial hypertension.

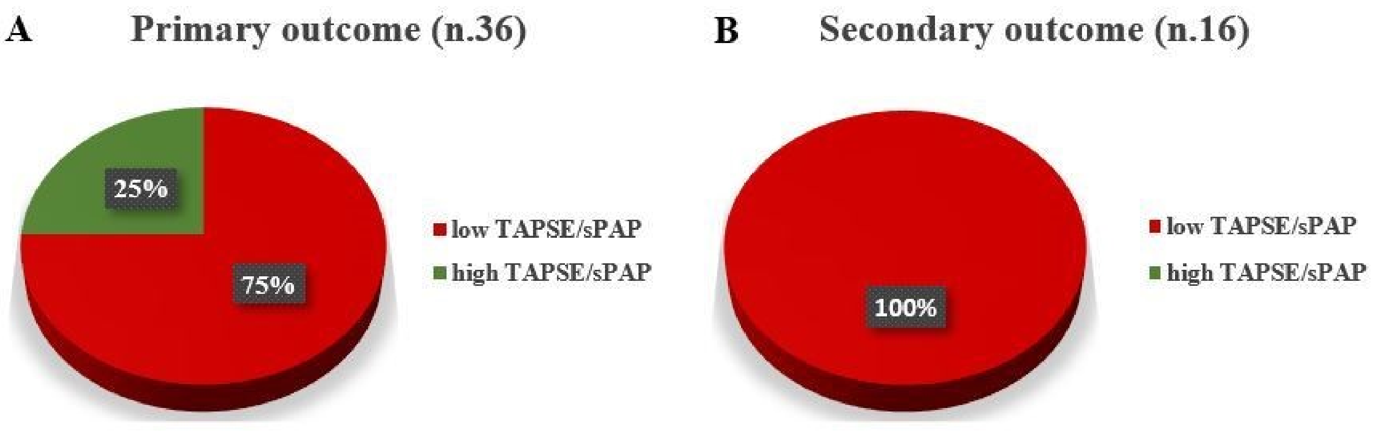

Supplementary Figure 3. Percentage of cumulative events for both primary and secondary outcomes stratified by TAPSE/sPAP ratio at six-year of follow-up.
Stratification by low TAPSE/sPAP ratio (<0.7 mm/mmHg) versus high TAPSE/sPAP ratio (≥0.7 mm/mmHg). A significantly higher proportion of all reported primary outcomes (75% of cardiac events) and secondary outcomes (100% of deaths) occurred in patients with a low TAPSE/sPAP ratio compared to patients with a high TASPE/sPAP ratio during the six years of follow up (p<0.001**).
The high TAPSE/sPAP ratio group showed no secondary outcomes. The threshold value of TAPSE/sPAP ratio 0.7 mm/mmHg derived from ROC analyses and the Youden Index.
ROC, receiver operating characteristic; sPAP, systolic pulmonary arterial pressure; TAPSE, tricuspid annular plane systolic excursion; **Mann-Whitney U-test.

Supplementary Table 1. Incremental prognostic value and model tests for TAPSE/sPAP ratio and NT-proANP for primary and secondary outcomes.

**Primary outcome**				
	X^2	*p*	**C-index**	**AIC**
**Model 1 (TAPSE/sPAP ratio)**	20.120	<0.001	0.75	29.485
**Model 2 (TAPSE/sPAP ratio and NT-proANP)**	25.876	<0.001	0.81	20.147
**Secondary outcome**				
	X^2	*p*	**C-index**	**AIC**
**Model 1 (TAPSE/sPAP ratio)**	23.012	<0.001	0.72	30.887
**Model 2 (TAPSE/sPAP ratio and NT-proANP)**	24.454	<0.001	0.85	15.844

AIC: Akaike information criterion; C: C-index; NT-proANP: amino-terminal atrial natriuretic peptide; sPAP: systolic pulmonary arterial pressure; TAPSE: tricuspid annular plane systolic excursion.
